# Poland syndrome accompanied by internal iliac artery supply disruption sequence: a case report

**DOI:** 10.1186/s13256-018-1823-8

**Published:** 2018-10-26

**Authors:** Kenji Gonda, Yosuke Tachiya, Yuichi Hatakeyama, Tomoyuki Momma, Tomoko Tamaoki, Yuko Maejima, Yuichi Rokkaku, Shigehira Saji, Kenju Shimomura, Koji Kono

**Affiliations:** 1 Department of Genetics, Fukushima Medical University, 1 Hikarigaoka, Fukushima City, Fukushima 960-1295 Japan; 2 Clinical Oncology Center, Fukushima Medical University Hospital, 1 Hikarigaoka, Fukushima City, Fukushima 960-1295 Japan; 3 Department of Gastrointestinal Tract Surgery, Fukushima Medical University, 1 Hikarigaoka, Fukushima City, Fukushima 960-1295 Japan; 4 Department of Surgery, Japan Community Healthcare Organization Nihonmatsu Hospital, 1-553 Naritamachi, Nihonmatsu City, Fukushima 964-8501 Japan; 5 Center of Genetics, Takatsuki Hospital, 1-3-13 Sobemachi, Takatsuki City, Osaka 569-1192 Japan; 6 Department of Pharmacology, Fukushima Medical University, 1 Hikarigaoka, Fukushima City, Fukushima 960-1295 Japan

**Keywords:** Poland syndrome, Internal iliac artery supply disruption, Pectoral muscle deficit, Renal hypoplasia

## Abstract

**Background:**

Poland syndrome is a congenital malformation characterized by ipsilateral hand and chest wall depression, including an absence or hypoplasia of the breast and pectoral muscles. These hypoplastic defects are reportedly caused by a subclavian artery supply disruption sequence.

**Case presentation:**

A 45-year-old Japanese woman, an out-patient, underwent an emergency examination for intense left lower abdominal pain. Computed tomography images revealed a hydronephrotic left kidney and dilatation of the left ureter. No ureteral calculus or neoplasm was found. In addition, no abnormalities connected to her left abdominal pain were found. Nephritis was diagnosed based on the results of urine analysis, and a course of antibiotics was administered. Computed tomography images also revealed a history of breast reconstruction with a custom-made silicone implant in her right breast. The present case showed symptoms of Poland syndrome, which were absence of the sternal head of the right pectoralis major and asymmetrical malformation of the chest wall due to hypoplasia of the right rib cage. In addition to typical Poland syndrome symptoms, she had hypoplasia of her right kidney, hypoplasia of the right gluteus minimus muscle, right-sided pelvic hypoplasia, spinal curvature to the right, and a cystic mass in her right ovary.

**Conclusions:**

In the present case of Poland syndrome, computed tomography images revealed malformation of the chest wall, absence of the pectoral muscle, and hypoplasia of a left kidney. Unilateral visceral hypoplasia is reported to be caused by a subclavian artery supply disruption sequence that occurs around 7 to 8 weeks of gestation. The present case can be considered a rare atypical phenotype of Poland syndrome with possible subclavian artery supply disruption sequence with internal iliac artery supply disruption.

## Background

PS (OMIM 173800) was originally described in 1841 by Alfred Poland presenting a 27-year-old patient with complete unilateral absence of the sternal head of the pectoralis major muscle and ipsilateral symbrachydactyly [1]. The Poland anomaly has an estimated incidence of 3–16 per 100,000 population, is more common among males (male-to-female ratio, 2 or 3:1), and reportedly manifests on the right side of the body in approximately 75% of the cases [2–4]. A range of other abnormalities have been recorded together with the Poland anomaly. These abnormalities include unilateral absence of the sternal head of the pectoralis major muscle [5], unilateral hypoplasia of the gluteus maximus in muscles in lower extremities [6], ipsilateral renal hypoplasia or agenesis [3, 4, 7–11], hypoplastic ribs [12], Sprengel’s deformity of the scapula [13], and phalangeal aplasia. These hypoplastic defects are reportedly caused by a subclavian artery supply disruption sequence (SASDS) and external iliac artery supply disruption, which occur in a similar period around the seventh to eighth week of gestation [14]. We report a rare case of an atypical phenotype of PS with possible hypoplasia of the right gluteus minimus muscle and novel internal iliac artery supply disruption.

## Case presentation

**Fig. 1:**
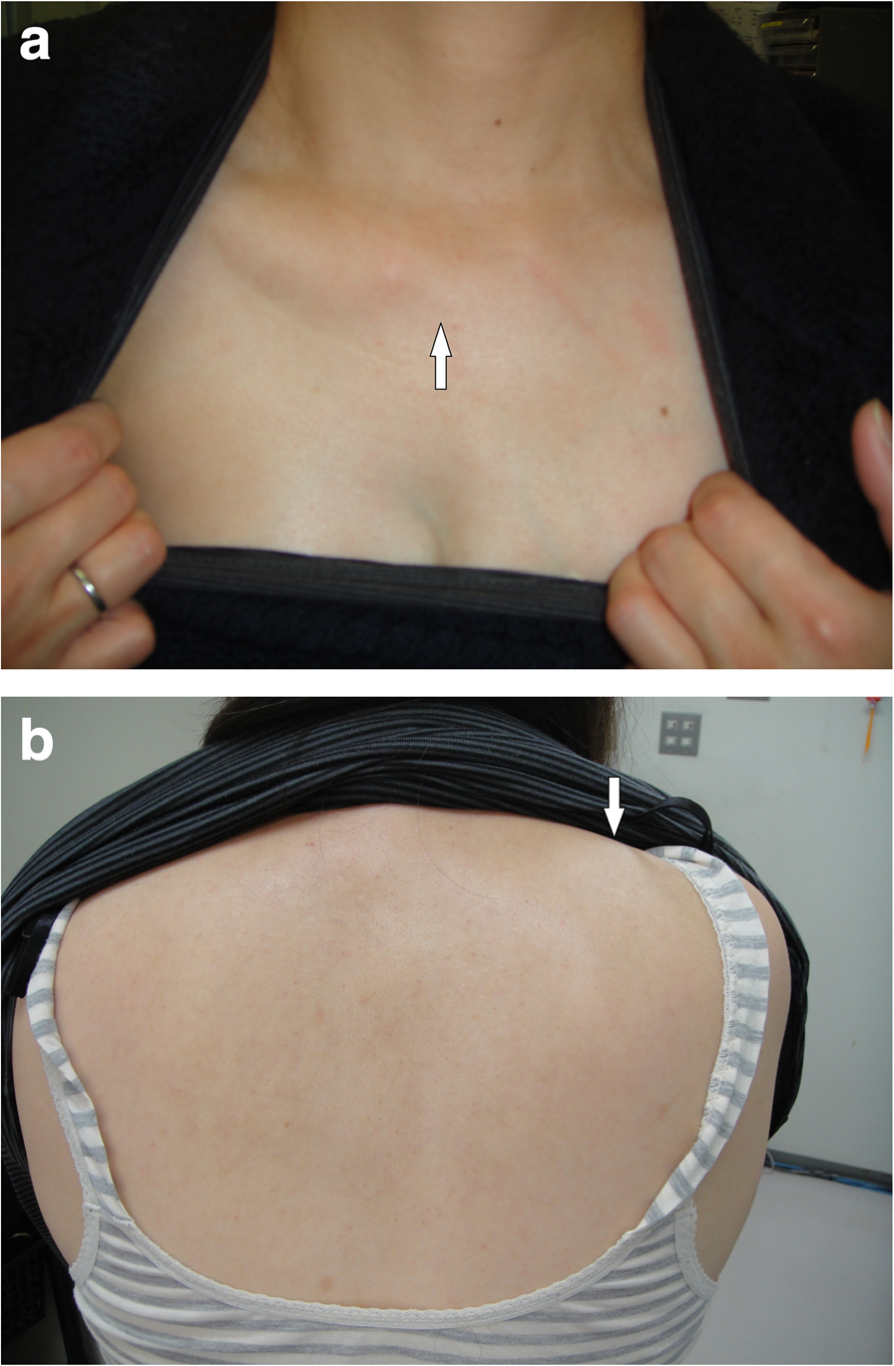
(**a**) Absence of the sternal head of the pectoralis major (white arrow) (**b**) Protrusion of the right scapula (white arrow)

A 45-year-old Japanese woman, an out-patient, underwent an emergency examination for left lower abdominal pain. She was in her usual state of good health until 1 day ago when she noticed the gradual onset of abdominal pain. Over the last 12–24 hours, she noticed it more in the left lower quadrant. Pain did not radiate, but stayed localized in the costovertebral angle (CVA) when her back was clapped. She had not noticed any fever, chills, or night sweats. She reported anorexia. She was not pregnant; she was not menstruating. She had no past medical history except for mammoplasty. No diseases run in her family. She drank alcohol occasionally; she did not smoke tobacco or use illegal drugs. She worked as an office worker. The physical findings were absence of the sternal head of the right pectoralis major (Fig. 1a) and protrusion of the right scapula (Fig. 1b). She reported right-sided weakness and presented symptoms of glaucoma. There were no symbrachydactyly on right hand and no signs of neurological disorders. She had been aware of an underdevelopment of one breast since infancy, lack of right underarm hair, difficulty exerting strength on the right side of her body, and feeling fatigue mainly on the right side when in a supine position.

**Fig. 2:**
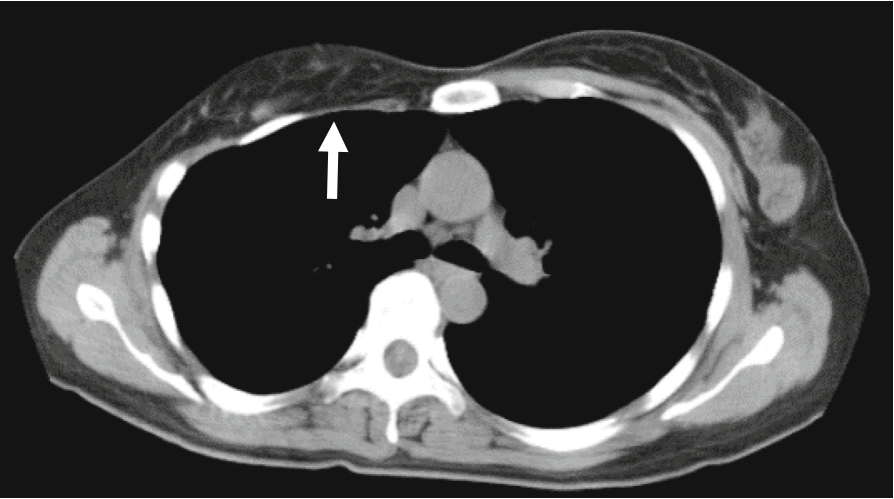
Defect of the pectoralis major (white arrow)

**Fig. 3:**
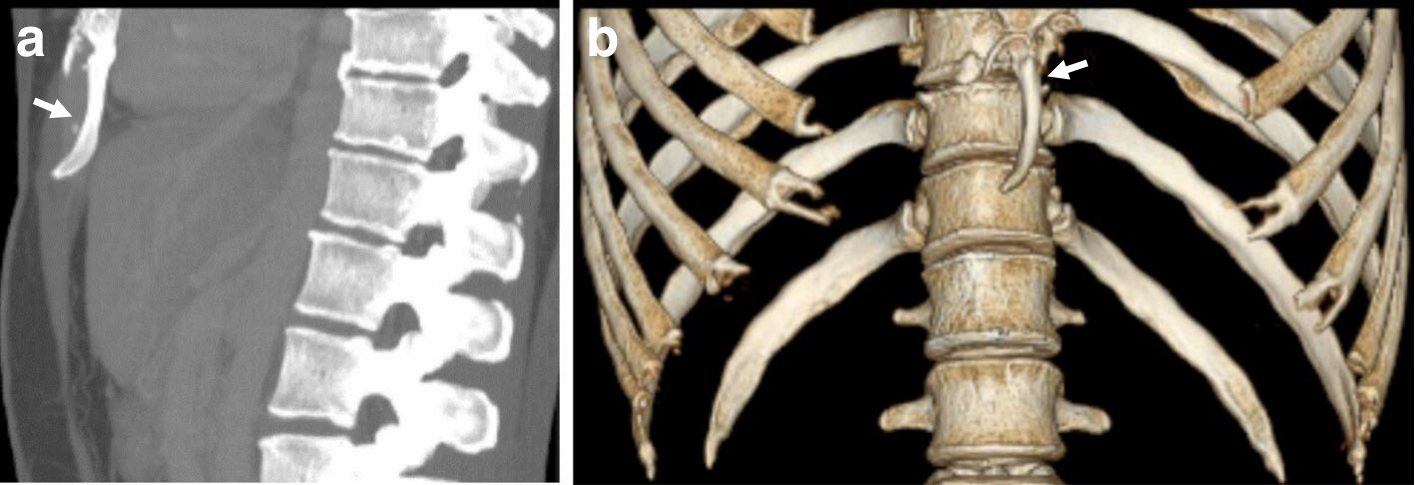
Anomaly of the xiphoid process (white arrow). **a** CT; **b** three-dimensional CT

**Fig. 4:**
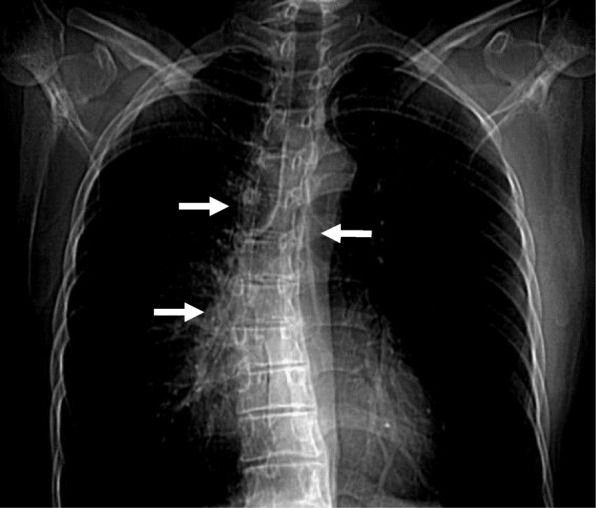
Curvature of the spine (white arrows)

**Fig. 5:**
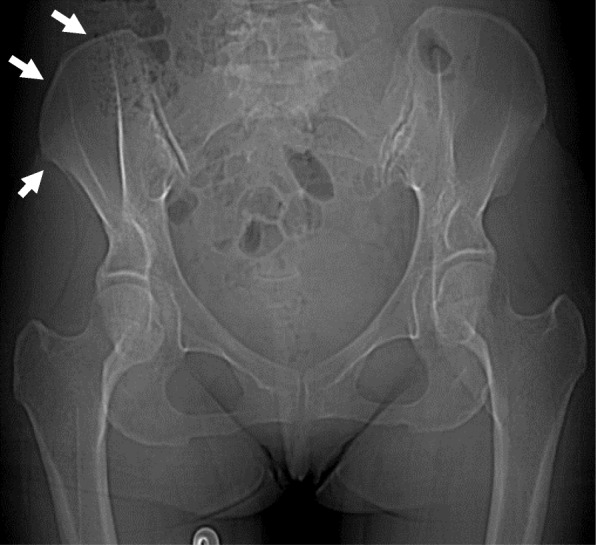
Hypoplasia of the right ilium (white arrows)

**Fig. 6:**
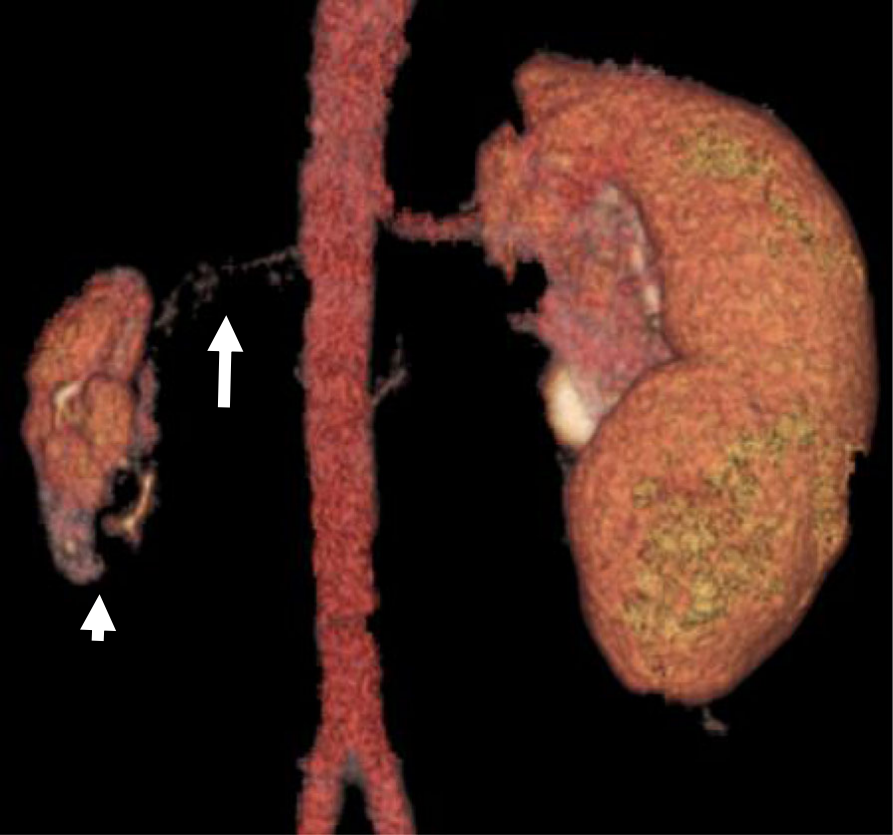
Hypoplasia of the right kidney (small white arrow) and renal artery (large white arrow)

**Fig. 7:**
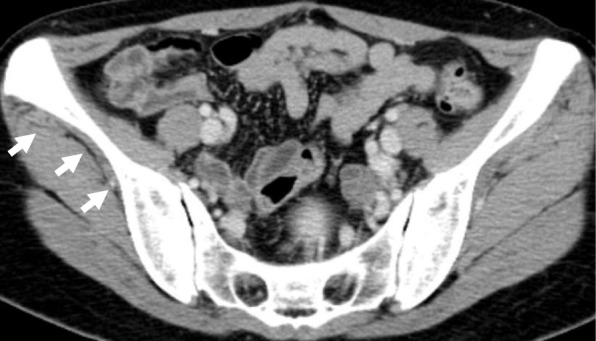
Hypoplasia of the right gluteus minimus (white arrows)

**Fig. 8:**
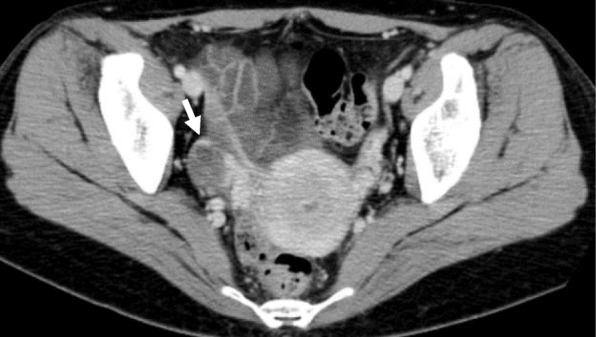
Right- sided ovarian cyst (white arrow)

**Fig. 9:**
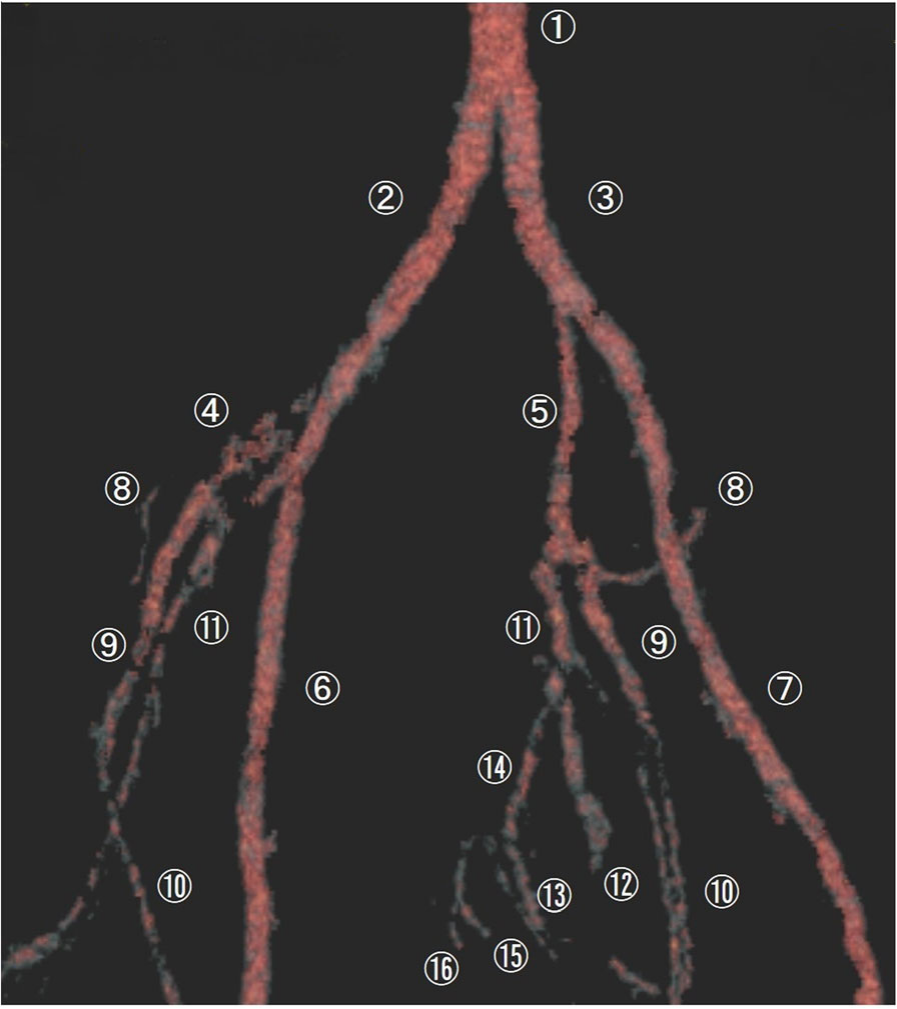
Hypoplasia of the right internal iliac artery. *1* abdominal aorta, *2* right common iliac artery, *3* left common iliac artery, *4* right internal iliac artery, *5* left internal iliac artery, *6* right external iliac artery, *7* left external iliac artery, *8* iliolumbar artery, *9* superior gluteal artery, *10* lateral sacral artery, *11* umbilical artery, *12* obturator artery, *13* inferior gluteal artery, *14* internal pudendal artery, *15* middle rectal artery, *16* inferior rectal artery

Computed tomography (CT) images revealed a hydronephrotic left kidney and dilatation of the left ureter. No ureteral calculus, neoplasm, or obstruction was found, and no abnormal findings that would explain her left abdominal pain were identified. Other CT findings were absence of the sternal head of the right pectoralis major, asymmetrical malformation of her chest wall due to hypoplasia of the right rib cage (Fig. 2), a shortened sternal body and anomaly of the xiphoid process (Fig. 3), spinal curvature to the right (Fig. 4), hypoplasia of the right ilium (Fig. 5), advanced atrophy of her right kidney (Fig. 6), hypoplasia of the right gluteus minimus (Fig. 7), and cystic mass in her right ovary (Fig. 8). Contrast-enhanced CT revealed a disruption of her right internal iliac artery (Fig. 9). Blood examinations revealed no abnormalities. Urine analysis revealed leukocytosis but no urinary blood. Nephritis was diagnosed and antibiotics were prescribed. She is currently showing improvement and receiving out-patient treatment. She has recently been on the verge of developing glaucoma and is seeing an ophthalmologist.

## Discussion and conclusions

PS is three times more common in males than females [15]; it is most commonly a sporadic condition [16] and no familial history is evident. Our present case (female) had no familial history for PS but showed widespread hypoplasia on the right side. However, unlike other reported cases so far, no unilateral digital hypoplasia or brachysyndactyly was found, and no concomitant Moebius syndrome [17] was evident. The cause of Poland anomalies with absence of the pectoral muscle but without hand abnormalities found in the present case is unclear.

Typically, PS is known to present reduced thoracic vasculature on the affected side [1]. The pathological mechanism in the development of PS is considered to be a result of interruption of the early embryonic blood supply to the subclavian artery [18], and this blood supply interruption is inherited as an autosomal dominant trait: OMIM 173800 [19]. A potential underlying genetic link between 10p13–14 duplication, PS, and congenital hyperinsulinemia is suggested [20]. In addition, PS has been reported in association with a *de novo* deletion of 11q12.3 in monozygotic twins [21]. However, the underlying genetic etiology of PS is not known.

Recent studies suggested an association of Moebius syndrome [17], Klippel–Feil syndrome, and Sprengel (congenital elevation of the scapula) anomalies with PS [14] and isolated absence of the pectoralis major with breast hypoplasia by investigating the embryonic development of the internal thoracic artery and cerebral arterial system. Each of these defects is suggested to result from the interruption of the early embryonic blood supply around the 37th to 42nd day of embryogenesis. This blood supply interruption was suggested to involve the subclavian artery, vertebral arteries, and their branches, and described as SASDS [14, 22, 23]. The present case presented isolated absence of the pectoralis major without limb defects possibly caused by the lack of sufficient blood supply to the subclavian and vertebral arteries during the embryonic stage.

Also, in the present case, CT images revealed hypoplasia of the right subclavian artery and curvature of the spine resulting from disruption of the vertebral arteries. Atrophy of the right gluteus minimus and hypoplasia of the right ilium were also observed. In previous studies, cases with a possible lower-extremity counterpart of the Poland sequence with unilateral hypoplasia of the gluteus maximus and pectoralis major were reported [6, 24, 25] with possible involvement of the disruption of the external iliac artery supply analogous to that of the subclavian artery supply. In the present case, the internal iliac artery was not bifurcated into the superior and inferior gluteal arteries, and blood appeared to be supplied by collateral circulation.

In addition, renal hypoplasia was observed in this case. A previous study reported a case of hypoplasia of the right pectoralis major and the right kidney caused by a fetal mesodermal defect [6, 11]. Our present case also showed atrophy of the right kidney possibly due to the disruption of the kidney arterial supply (Fig. 6) [11, 26]. As for the ovarian cyst found in this case, the underdevelopment of the Müllerian ducts due to an internal iliac artery supply disruption formed at 7 to 8 weeks of gestation may be a possible mechanism for its development.

Our present case indicates the importance of screening for internal organ anomalies when encountering patients with PS. In this case study, we have described a patient with PS with renal hypoplasia and atrophy of the gluteus minimus. Such anomalies have reportedly been caused by SASDS and external iliac artery supply disruption in the upper and lower body, respectively, occurring at a similar time around the seventh to eighth fetal week. Here we present a rare case that can be regarded as synchronous SASDS and internal iliac artery supply disruption. She brings up a child alone and is not rich enough economically to have a genetic test. We asked her to go to a hospital again if she had abdominal pain. She may receive artificial kidney dialysis in future, as the function of her kidney decreases. Therefore we are going to submit an application to local government so that national and local governments provide subsidies to patients with PS who have genetic disease like this patient.

## Abbreviations

CT: Computed tomography
OMIM: Online Mendelian Inheritance in Man
PS: Poland syndrome
SASDS: Subclavian artery supply disruption sequence

## References

[1] Poland A. Deficiency of the pectoral muscles. Guy’s Hosp Rep. 1841;6:191.

[2] Freire-Maia N, Chautard EA, Opitz JM, Freire-Maia A, Quelce-Salgado A. The Poland syndrome-clinical and genealogical data, dermatoglyphic analysis, and incidence. Hum Hered. 1973;23(2):97–104. 10.1159/000152560 4356989

[3] Miller RA, Miller DA. Letter: Congenital absence of the pectoralis major muscle with acute lymphoblastic leukemia and genitourinary anomalies. J Pediatr. 1975;87(1):146–7. 10.1016/s0022-3476(75)80095-3 239106

[4] Temtamy SA, McKusick VA. The genetics of hand malformations. Birth Defects Orig Artic Ser. 1978;14:1–619. 215242

[5] David TJ, Winter RM. Familial absence of the pectoralis major, serratus anterior, and latissimus dorsi muscles. J Med Genet. 1985;22(5):390–2. 10.1136/jmg.22.5.390 PMC1049486 4078869

[6] Riccardi VM. Unilateral gluteal hypoplasia and brachysyndactyly: lower extremity counterpart of the Poland anomaly. Pediatrics. 1987;61(4):653–4. 208046

[7] Assadi FK, Salem M. Poland syndrome associated with renal agenesis. Pediatr Nephrol. 2002;17(4):269–71. 10.1007/s00467-001-0804-z 11956880

[8] Curran AS, Curran JP. Associated acral and renal malformations: a new syndrome? Pediatrics. 1972;49(5):716–25. 4402497

[9] Hegde HR, Leung AK. Aplasia of pectoralis major muscle and renal anomalies. Am J Med Genet. 1989;32(1):109–11. 10.1002/ajmg.1320320123 2650545

[10] Mace JW, Kaplan JM, Schanberger JE, Gotlin RW. Poland’s syndrome. Report of seven cases and review of the literature. Clin Pediatr (Phila). 1972;11(2):98–102. http://journals.sagepub.com/doi/10.1177/000992287201100217 10.1177/000992287201100217 4337259

[11] Qvist N, Nielsen K, Christensen PV. Aplasia of major pectoral muscle combined with renal aplasia and cystic malformation of common iliac vein. Urology. 1987;29(4):434–5. 10.1016/0090-4295(87)90519-x 3564221

[12] Tomos I, Papaioannou AI, Vlami A, Apollonatou V, Manali ED, Papiris SA. Unilateral hypertransparency on chest radiograph: the congenital Poland syndrome. Adv Respir Med. 2016;84(6):342–3. https://journals.viamedica.pl/advances_in_respiratory_medicine/article/view/48813 10.5603/ARM.2016.0045 28009035

[13] Ireland DC, Takayama N, Flatt AE. Poland’s syndrome. J Bone Joint Surg Am. 1976;58:52–8. 175070

[14] Bavinck JN, Weaver DD. Subclavian artery supply disruption sequence: hypothesis of a vascular etiology for Poland, Klippel-Feil, and Möbius anomalies. Am J Med Genet. 1986;23(4):903–18. http://onlinelibrary.wiley.com/doi/10.1002/ajmg.1320230405/abstract;jsessionid=554DE27A04C89618A34680A741AFAD89.f02t03 10.1002/ajmg.1320230405 3008556

[15] McGillivray BC, Lowry RB. Poland syndrome in British Columbia: incidence and reproductive experience of affected persons. Am J Med Genet. 1977;1(1):65–74. http://onlinelibrary.wiley.com/doi/10.1002/ajmg.1320010108/abstract 10.1002/ajmg.1320010108 205132

[16] David TJ. Familial Poland anomaly. J Med Genet. 1982;19(4):293–6. 10.1136/jmg.19.4.293 PMC1048897 6288948

[17] Sugarman GI, Stark HH. Möbius syndrome with Poland’s anomaly. J Med Genet. 1973;10(2):192–6. https://www.ncbi.nlm.nih.gov/pmc/articles/PMC1013017/10.1136/jmg.10.2.192 PMC1013017 4351696

[18] Bouvet JP, Leveque D, Bernetieres F, Gros JJ. Vascular origin of Poland syndrome? A comparative rheographic study of the vascularisation of the arms in eight patients. Eur J Pediatr. 1978;128(1):17–26. 10.1007/BF00496922 208850

[19] Fraser FC, Ronen GM, O’Leary E. Pectoralis major defect and Poland sequence in second cousins: extension of the Poland sequence spectrum. Am J Med Genet. 1989;33(4):468–70. http://onlinelibrary.wiley.com/doi/10.1002/ajmg.1320330409/abstract 10.1002/ajmg.1320330409 2556919

[20] Giri D, Patil P, Hart R, Didi M, Senniappan S. Congenital hyperinsulinism and Poland syndrome in association with 10p13-14 duplication. Endocrinol Diabetes Metab Case Rep. 2017. https://www.edmcasereports.com/articles/endocrinology-diabetes-and-metabolism-case-reports/10.1530/EDM-16-0125. 10.1530/EDM-16-0125 PMC5404473 28458900

[21] Vaccari CM, Romanini MV, Musante I, Tassano E, Gimelli S, Divizia MT, Torre M, Morovic CG, Lerone M, Ravazzolo R, Puliti A. *De novo* deletion of chromosome 11q12.3 in monozygotic twins affected by Poland syndrome. BMC Med Genet. 2014;15:63. https://bmcmedgenet.biomedcentral.com/articles/10.1186/1471-2350-15-63 10.1186/1471-2350-15-63 PMC4051386 24885342

[22] Charles I, Scott J. Pectoral girdle, spine, ribs, and pelvic girdle, human malformations and related anomalies, vol. 2. New York: Oxford University Press; 1993. p. 655–97.

[23] David TJ. Nature and etiology of the Poland anomaly. N Engl J Med. 1972;287(10):487–9. 10.1056/NEJM197209072871004 4340236

[24] Corona-Rivera JR, Corona-Rivera A, Totsuka-Sutto SE, Corona-Rivera E. Corroboration of the lower extremity counterpart of the Poland sequence. Clin Genet. 1997;51(4):257–9. 10.1111/j.1399-0004.1997.tb02465.x 9184248

[25] Parano E, Falsaperla R, Pavone V, Toscano A, Bolan EA, Trifiletti RR. Intrafamilial phenotypic heterogeneity of the Poland complex: a case report. Neuropediatrics. 1995;26(4):217–9. https://www.thieme-connect.de/DOI/DOI?10.1055/s-2007-979758 10.1055/s-2007-979758 8544963

[26] Gadoth N, Biedner B, Torok G. Mobius syndrome and Poland anomaly: case report and review of the literature. J Pediatr Ophthalmol Strabismus. 1979;16(6):374–6. 10.3928/0191-3913-19791101-09 521879

